# LPS Induces Occludin Dysregulation in Cerebral Microvascular Endothelial Cells via MAPK Signaling and Augmenting MMP-2 Levels

**DOI:** 10.1155/2015/120641

**Published:** 2015-07-28

**Authors:** Lan-hui Qin, Wen Huang, Xue-an Mo, Yan-lan Chen, Xiang-hong Wu

**Affiliations:** ^1^Department of Neurology, First Affiliated Hospital, Guangxi Medical University, Nanning 530021, China; ^2^Department of Vasculocardiology, First Affiliated Hospital, Guangxi Medical University, Nanning 530021, China

## Abstract

Disrupted blood-brain barrier (BBB) integrity contributes to cerebral edema during central nervous system infection. The current study explored the mechanism of lipopolysaccharide- (LPS-) induced dysregulation of tight junction (TJ) proteins. Human cerebral microvascular endothelial cells (hCMEC/D3) were exposed to LPS, SB203580 (p38MAPK inhibitor), or SP600125 (JNK inhibitor), and cell vitality was determined by MTT assay. The proteins expressions of p38MAPK, JNK, and TJs (occludin and zonula occludens- (ZO-) 1) were determined by western blot. The mRNA levels of TJ components and MMP-2 were measured with quantitative real-time polymerase chain reaction (qRT-PCR), and MMP-2 protein levels were determined by enzyme-linked immunosorbent assay (ELISA). LPS, SB203580, and SP600125 under respective concentrations of 10, 7.69, or 0.22 *µ*g/mL had no effects on cell vitality. Treatment with LPS decreased mRNA and protein levels of occludin and ZO-1 and enhanced p38MAPK and JNK phosphorylation and MMP-2 expression. These effects were attenuated by pretreatment with SB203580 or SP600125, but not in ZO-1 expression. Both doxycycline hyclate (a total MMP inhibitor) and SB-3CT (a specific MMP-2 inhibitor) partially attenuated the LPS-induced downregulation of occludin. These data suggest that MMP-2 overexpression and p38MAPK/JNK pathways are involved in the LPS-mediated alterations of occludin in hCMEC/D3; however, ZO-1 levels are not influenced by p38MAPK/JNK.

## 1. Introduction

Central nervous system (CNS) microenvironment homeostasis is essential for normal function and is maintained by the blood-brain barrier (BBB). This critical structure's integrity is influenced by the cerebral microvasculature, cellular transport pathways, enzymatic machinery, and human cerebral microvascular endothelial cells interconnected by tight junctions (TJs) [1]. It has been shown that brain endothelial cells are a primary target of immunological attacks during bacterial infections associated with TJ disruption [2]. TJ proteins play a key role in restricting paracellular pathway permeability, and their destruction increases diffusion across the BBB. Among different TJ proteins, occludin and ZO-1 are heavily involved in regulating BBB permeability [3–5]. Occludin was the first integral membrane protein identified within endothelial cell TJs [6], and recessive mutations in the gene encoding occludin have been shown to lead to abnormal cortical development [7]. Occludin directly determines paracellular permeability to different ions or large molecules [8]. Importantly, ZO-1 is required for appropriate occludin localization at TJs [9]. ZO-1 is a member of the membrane-associated guanylate kinase homologue family of proteins and is also a critical regulator of TJ assembly. The Tsukita group recently demonstrated that depletion of both ZO-1 and ZO-2 in the EpH4 mammary epithelial cell line completely abrogated TJs assembly [10]. ZO-1 redistribution is the earliest cellular event during cell shedding and may be a key event in inflammatory diseases [11]. These findings indicate that the TJ proteins occludin and ZO-1 play important roles in regulating BBB permeability. Previous studies have suggested that matrix metalloproteinases (MMPs) play a critical role in BBB disruption [12]. Therefore, measuring MMP expression and activity and finding pathways that influence changes in these parameters may lead to more effective BBB protection therapies and improved treatments for CNS infection.

Stress-activated mitogen-activated protein kinase (MAPK) signaling pathways have been recognized as critical factors of neuroinflammation in human brain microvascular endothelial cells [13]. Extracellular-signal-regulated kinase (ERK) 1/2 and Akt are involved in the regulatory mechanisms of peroxisome proliferator-activated receptor- (PPAR-) mediated protection against HIV-1-induced MMP-9 expression [13]. Lipopolysaccharide (LPS) is a major component of the cell walls of Gram-negative bacteria that can infect the CNS [14]. BBB dysfunction following LPS administration has been observed and confirmed in numerous studies [15, 16]. At the structural level, BBB dysfunction is linked to alterations of TJ structure and function. LPS induces redistribution of ZO-1 and occludin in human umbilical vein endothelial cells and Caco2 cells via its effects on the I*κ*B*α* or ERK-MAPK pathway [4, 17]. However, whether p38MAPK and c-Jun N-terminal kinase (JNK) signaling pathways are involved in TJ protein disruption is still unknown. Understanding the mechanisms regulating TJ protein expression may provide insight into developing therapeutic tools to prevent BBB damage and subsequent influx of inflammatory cells into the CNS. An immunofluorescence study of TJ proteins revealed that the normal linear patterns of the ZO-1 and occludin signals at the cell margins shifted to zipper-like or zigzag shapes following LPS treatment [4, 18]. Therefore, the aim of the present study was to evaluate the roles of p38MAPK and JNK signaling in LPS-induced TJ protein disruption in human cerebral microvascular endothelial cells (hCMEC/D3).

Our results indicated that LPS altered occludin and ZO-1 expressions, but only the decreases in occludin mRNA and protein expressions were reversed by inhibiting phosphorylation of p38MAPK and JNK signaling pathways. Moreover, p38MAPK and JNK signaling pathways mediated LPS-induced MMP-2 overexpression in hCMEC/D3, and both MMP-2 and total MMP inhibitors attenuated the LPS-induced downregulation of occludin.

## 2. Materials and Methods

### 2.1. Cell Cultures

hCMEC/D3 maintain the overall TJ organization known to be present in the brain endothelium making them an excellent model for BBB function studies [19]; they were kindly provided by Pierre-Olivier Couraud (INSERM, France). The cells were seeded on rat tail collagen-coated 6-well plates and cultured in EBM-2 supplemented with 1% penicillin-streptomycin, 1.4 *μ*M hydrocortisone, 5 *μ*g mL^−1^ ascorbic acid, 1/100 chemically defined lipid concentrate, 10 mM HEPES, 1 ng mL^−1^ basic fibroblast growth factor (bFGF), and 5% fetal bovine serum as recommended by the manufacturer (Lonza, Switzerland). The cells were maintained at 37°C in a humidified atmosphere of 5% CO_2_. Culture medium was changed every 1 to 2 days.

### 2.2. Cell Viability

We used 3-(4,5-dimethylthiazol-2-yl)-2,5-diphenyltetrazolium bromide (MTT; Solarbio, Beijing, China) assays to measure the viability of cells cultured in the 96-well culture plates at a density of 1 × 10^4^ cells/well and treated with LPS or SB203580 (p38MAPK inhibitor, Cell Signaling Technology, Danvers, MA, USA) or SP600125 (JNK inhibitor, Sigma, St. Louis, MO, USA) for 24 h at various concentrations.

### 2.3. Western Blot Analysis

Treated endothelial cultures were washed three times with ice-cold phosphate-buffered saline before proteins were extracted with RIPA buffer (Beyotime, Jiangsu, China) containing protease and phosphatase inhibitor cocktail tablets. Cell lysates were centrifuged (12,000 ×g at 4°C for 15 min), and the supernatants were collected and determined with BCA protein assay kits (Beyotime). Total proteins were mixed with 5x SEMS-PAGE protein sample buffer (Beyotime), then boiled for 5 min at 100°C, and stored at −20°C until use. Equal masses of proteins (30 *μ*g) were separated by SDS-PAGE and transferred to polyvinylidene fluoride (PVDF) membranes (0.22/0.45 *μ*m, Millipore, Billerica, MA, USA). The membranes were blocked for 1.5 hours with 5% milk in TBST (0.1% Tween-20 in tris-buffered saline) at room temperature and incubated overnight at 4°C with primary antibodies against p-p38, total p38MAPK, p-JNK, and total JNK (1 : 1000, Cell Signaling Technology); occludin (1 : 1500; Invitrogen, Carlsbad, CA, USA), ZO-1 (1 : 1500; Epitomics, Burlingame, CA, USA), and GAPDH (1 : 10,000; Proteintech Group, Chicago, IL, USA). The membranes were further incubated with horseradish peroxidase- (HRP-) conjugated secondary antibody (goat anti-mouse purchased from MultiSciences, Hangzhou, China; goat anti-rabbit purchased from SAB, College Park, MD, USA; 1 : 8000) for 1 h at room temperature and finally developed with an electrochemiluminescence system (ECL; APPLYGEN, Beijing, China). To quantify relative protein expression levels, the intensity of specific protein bands was quantified using ImageJ software (National Institutes of Health, Bethesda, MD, USA) and then normalized to the level of the respective loading control protein.

### 2.4. Reverse Transcription and Quantitative Real-Time PCR (qRT-PCR)

Total RNA was isolated from treated endothelial cells using TRIzol reagent (Invitrogen). Then, 1 *μ*g total RNA was reverse transcribed with the PrimeScript RT reagent kit (Takara Bio, Dalian, Japan) according to the manufacturer's instructions. The sequences of primers (Invitrogen) were as follows: occludin, 5′-TCAGGGAATATCCACCTATCACTTCAG-3′ and 5′-CATCAGCAGCAGCCATGTACTCTTCAC-3′; ZO-1, 5′-GTGCCAGGAAGTTATACGAGCG-3′ and 5′-CACCATACCAACCATCATTCATTG-3′; MMP-2, 5′-CGTCTGTCCCAGGATGACATC-3′ and 5′-TGTCAGGAGAGGCCCCATAG; GAPDH, 5′-GCACCGTCAAGGCTGAGAAC-3′ and 5′-TGGTGAAGACGCCAGTGGA-3′. Quantitative RT-PCR was performed with a Taq PCR Master Mix kit (Takara Bio, Dalian, Japan) using the ABI Prism 7300 Sequence Detection System (Applied Biosystems, Foster City, CA, USA). PCR cycles consisted of an initial denaturation step at 95°C for 30 s, followed by 95°C for 5 s, and 60°C for 31 s. All values were calculated using the ΔΔCt method and expressed as the change relative to GAPDH mRNA expression.

### 2.5. Enzyme-Linked Immunosorbent Assay (ELISA)

Culture supernatants were harvested from treated endothelial cells, centrifuged to remove cellular debris, and stored at −80°C. MMP-2 protein levels were measured with a Human MMP-2 ELISA kit (Abcam, Cambridge, UK) according to the manufacturer's instructions. Each ELISA was carried out in duplicate for at least three separate experiments.

### 2.6. Statistical Analysis

Standard statistical analysis was completed using SPSS16.0 (SPSS, Chicago, IL, USA). Differences between groups were analyzed using one-way analysis of variance (ANOVA). *P* < 0.05 was considered significant.

## 3. Results

### 3.1. Cell Viability

**Figure 1:**
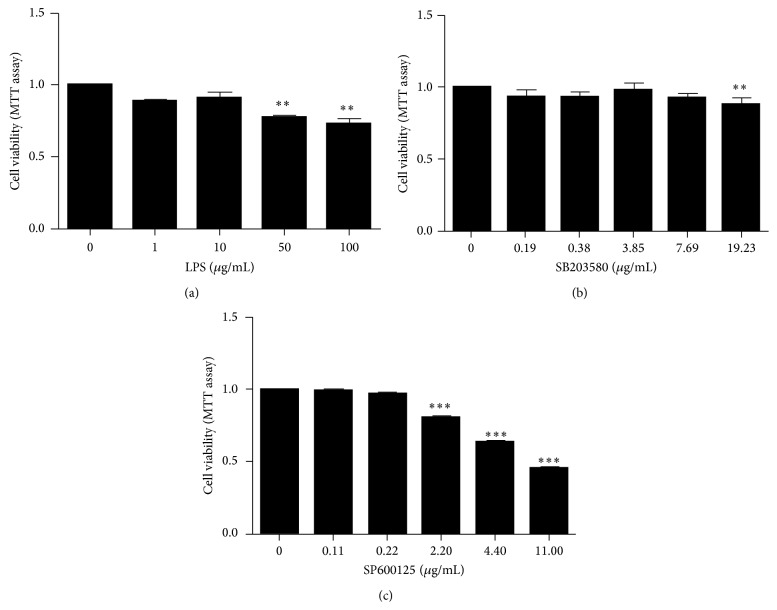
Cell viability. hCMEC/D3 were treated with different concentrations of LPS (0, 1, 10, 50, and 100 *μ*g/mL), SB203580 (0, 0.19, 0.38, 3.85, 7.69, and 19.23 *μ*g/mL), or SP600125 (0, 0.11, 0.22, 2.20, 4.40, and 11.00 *μ*g/mL) for 24 h. Cell viability was not affected by LPS, SB203580, and SP600125 concentrations under 10, 7.69, and 0.22 *μ*g/mL, respectively. The results are the mean ± SEM (*n* = 3). ^*∗∗*^ *P* < 0.01, ^*∗∗∗*^ *P* < 0.001 versus control.

hCMEC/D3 were treated with different concentrations of LPS (0, 1, 10, 50, and 100 *μ*g/mL), SB203580 (0, 0.19, 0.38, 3.85, 7.69, and 19.23 *μ*g/mL), or SP600125 (0, 0.11, 0.22, 2.20, 4.40, and 11.00 *μ*g/mL) for 24 h. Cell viability was not affected by LPS, SB203580, and SP600125 concentrations under 10, 7.69, and 0.22 *μ*g/mL, respectively (Figure 1).

### 3.2. LPS Decreases Occludin and ZO-1 Protein Expression

**Figure 2:**
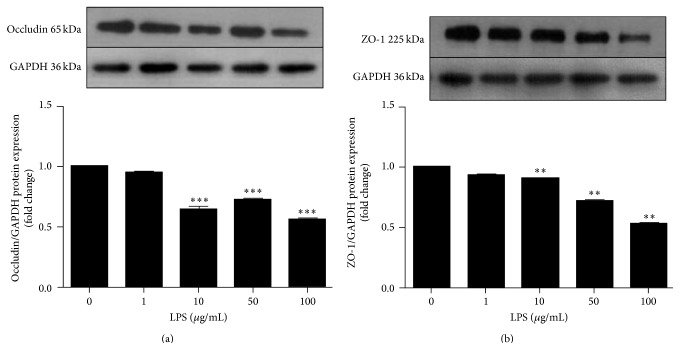
Effects of LPS on TJs expression. hCMEC/D3 were exposed to different concentrations of LPS (0, 1, 10, 50, and 100 *μ*g/mL) for 24 h. Protein levels of occludin and ZO-1 were detected by western blot. The relative intensities of occludin and ZO-1 were calculated as a ratio of target protein to GAPDH. Quantification of occludin (a) and ZO-1 (b) expressions is shown as a bar graph. Results are the mean ± SEM (*n* = 3). ^*∗∗*^ *P* < 0.01, ^*∗∗∗*^ *P* < 0.001 versus control.

To examine the effects of LPS on the TJ protein expression levels, hCMEC/D3 were exposed to different concentrations of LPS (0, 1, 10, 50, and 100 *μ*g/mL) for 24 h (Figure 2). LPS treatment significantly decreased occludin protein levels at concentrations of 10, 50, and 100 *μ*g/mL. LPS induced ZO-1 downregulation in a dose-dependent fashion for concentrations of 10, 50, and 100 *μ*g/mL.

### 3.3. p38MAPK and JNK Signaling Are Involved in LPS-Induced Changes in Occludin but Not ZO-1 Expression

**Figure 3:**
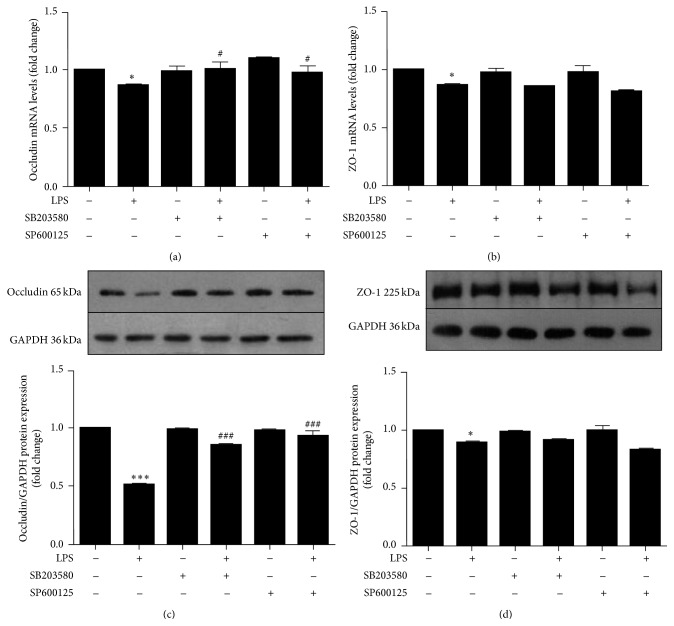
Roles of p38MAPK and JNK signaling in LPS-induced alterations of occludin and ZO-1. hCMEC/D3 were pretreated with inhibitors of p38MAPK (SB203580, 3.85 *μ*g/mL) or JNK signaling pathways (SP600125, 0.22 *μ*g/mL) for 1 h prior to 24-hour LPS treatment (10 *μ*g/mL). Protein levels of occludin (a) and ZO-1 (b) were detected by western blot. Occludin (c) and ZO-1 (d) RNA levels were measured with qRT-PCR. Data are expressed as mean ± SEM (*n* = 3). ^*∗*^ *P* < 0.05 and ^*∗∗∗*^ *P* < 0.001 versus control. ^#^ *P* < 0.05, ^###^ *P* < 0.001 versus LPS.

To explore whether the p38MAPK and JNK signaling are involved in LPS-induced downregulation of occludin and ZO-1, cells were treated with p38MAPK inhibitor (SB203580, 3.85 *μ*g/mL) or JNK inhibitor (SP600125, 0.22 *μ*g/mL) 1 h before LPS treatment. Both SB203580 and SP600125 partially attenuated occludin downregulation at the mRNA (Figure 3(a)) and protein (Figure 3(c)) levels compared with the LPS-treated group (*P* < 0.05 and *P* < 0.001, resp.). Conversely, no changes were noted for ZO-1 (*P* > 0.05, Figures 3(b) and 3(d)).

### 3.4. LPS Enhances p38MAPK and JNK Phosphorylation

**Figure 4:**
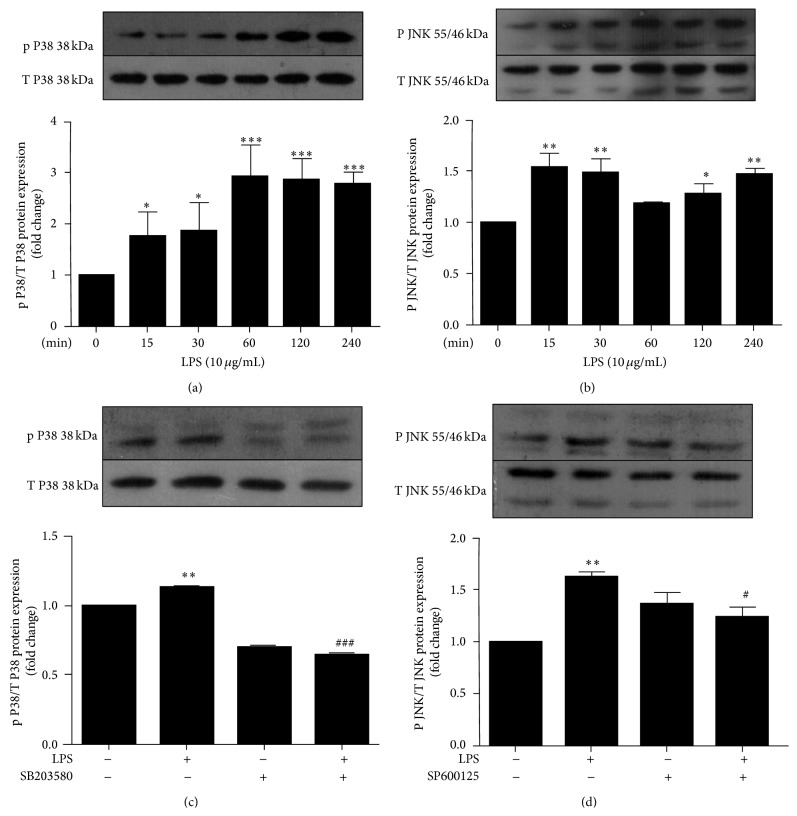
LPS activates p38MAPK and JNK phosphorylation. hCMEC/D3 were incubated with 10 *μ*g/mL LPS for different time periods (0, 15, 30, 60, 120, and 240 min) before phosphorylation levels of p-p38 (a) and p-JNK (b) were tested by western blot. Next, hCMEC/D3 were pretreated with SB203580 (3.85 *μ*g/mL) or SP600125 (0.22 *μ*g/mL) 1 h prior to 1-hour LPS exposure (10 *μ*g/mL), and phosphorylation levels of p-p38 (c) and p-JNK (d) were detected by western blot. Results are the mean ± SEM (*n* = 3). ^*∗*^ *P* < 0.05, ^*∗∗*^ *P* < 0.01, and ^*∗∗∗*^ *P* < 0.001 versus control. ^#^ *P* < 0.05, ^###^ *P* < 0.001 versus LPS.

To determine whether LPS augments p38 and JNK phosphorylation in hCMEC/D3, p-p38 and p-JNK expression levels were tested by western blot analysis (Figure 4). When cells were treated with 10 *μ*g/mL LPS for different time periods (0, 15, 30, 60, 120, and 240 min), p-p38 and p-JNK levels gradually increased and peaked at 60 and 15 min, respectively (Figures 4(a) and 4(b)). Phosphorylation of p38 and JNK pathways in hCMEC/D3 was significantly abrogated by their respective inhibitors, SB203580 and SP600125 (Figures 4(c) and 4(d)). The data suggest that LPS probably induces the destruction of TJ proteins by regulating the phosphorylation (activity) of p38MAPK and JNK.

### 3.5. LPS Increases MMP-2 Expression via p38MAPK and JNK Signaling Pathways

**Figure 5:**
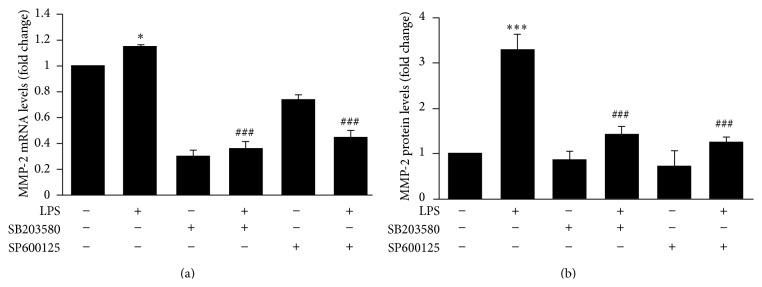
p38MAPK and JNK signaling pathways involved in LPS-induced alterations in MMP-2 mRNA and protein levels. Following treatment with inhibitors of p38MAPK and JNK signaling pathways, MMP-2 mRNA and protein levels were detected by qRT-PCR and ELISA, respectively. Results are mean ± SEM (*n* = 3). ^*∗*^ *P* < 0.05, ^*∗∗∗*^ *P* < 0.001 versus control. ^###^ *P* < 0.001 versus LPS.

Recent studies have indicated that MMPs, especially MMP-2, play a critical role in BBB disruption [12, 13]. To determine whether p38MAPK and JNK signaling pathways are involved in LPS-induced MMP-2 production, hCMEC/D3 were pretreated with SB203580 (3.85 *μ*g/mL) or SP600125 (0.22 *μ*g/mL) before LPS (10 *μ*g/mL) treatment. LPS significantly activated MMP-2 as detected by real-time PCR (Figure 5(a)) and ELISA (Figure 5(b)); however, both inhibitors attenuated the LPS-induced increase in MMP-2 expression (Figures 5(a) and 5(b)).

### 3.6. MMP-2 Is Involved in LPS-Induced Occludin Expression

**Figure 6:**
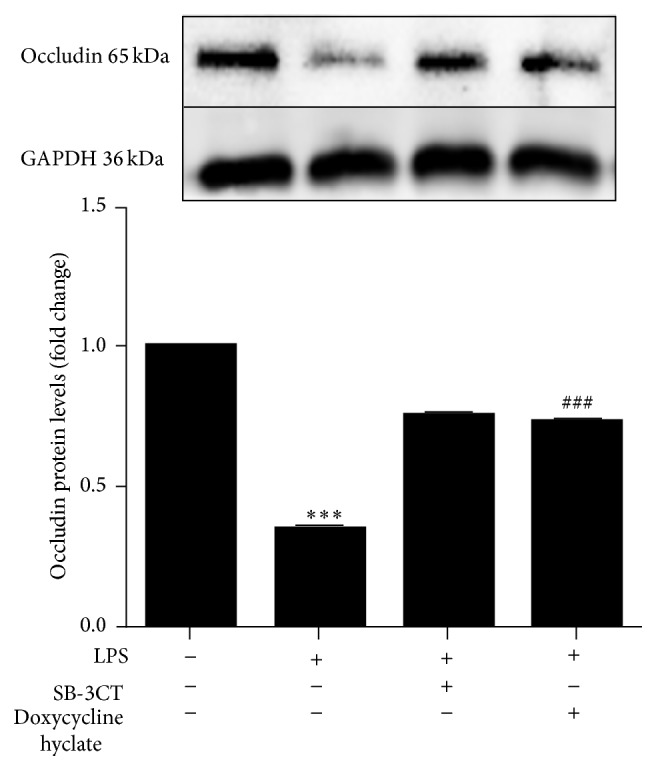
Effects of MMP-2 on LPS-induced occludin expression. hCMEC/D3 were pretreated with inhibitors of total MMPs (doxycycline hyclate 1 *μ*g/mL) or MMP-2 (SB-3CT 13.9 nmol/L) for 1 h prior to 24-hour LPS treatment (10 *μ*g/mL). Occludin protein levels were detected by western blot. Data are expressed as mean ± SEM (*n* = 3). ^*∗∗∗*^ *P* < 0.001 versus control. ^###^ *P* < 0.001 versus LPS.

To further explore the mechanism of LPS-mediated occludin disruption and confirm whether MMPs and especially MMP-2 affect occludin expression, cells were pretreated with inhibitors of total MMPs (doxycycline hyclate, 1 *μ*g/mL) or MMP-2 (SB-3CT, 13.9 nmol/L). Both inhibitors partially attenuated the LPS-induced downregulation of occludin as detected by western blot (*P* < 0.001, Figure 6).

## 4. Discussion

BBB integrity is compromised during CNS infection, increasing permeability and strongly contributing to secondary brain edema, which directly influences patient prognosis. Studies have shown that occludin and ZO-1 play important roles in regulating BBB permeability [3, 4]. mRNA transcription and protein expression of TJ-associated proteins (claudin-5, occludin, and ZO-1) are significantly reduced following traumatic brain injury, and these changes are consistent with greater BBB permeability [6]. BBB destruction allows blood-borne immune cells to enter the CNS and elicit neuroinflammatory responses [16].

Increased endothelial cell permeability can occur following stimulation by a variety of inflammatory mediators including LPS, an endotoxin found in the outer membrane of Gram-negative bacteria that stimulates mononuclear cells and neutrophils to secrete immunoregulatory and proinflammatory cytokines [20]. Transendothelial electrical resistance (TEER) indicates BBB integrity, and previous studies have demonstrated that LPS increases the paracellular permeability of brain endothelial cells and decreases TEER [21–23]. This may allow pathogenic bacteria to cross the BBB and accelerate disease processes. LPS treatment of hCMEC/D3 suppresses mRNA expression of claudin-5/occludin as well as protein levels of claudin-5/ZO-1 [24]. Our results demonstrate that LPS (10, 50, and 100 *μ*g/mL) obviously decreases occludin and ZO-1 protein levels (Figure 2). Moreover, LPS (10 *μ*g/mL) did not affect cell viability (Figure 1(a)). Indeed, a previous study reported that LPS significantly increased BBB integrity at 8 h and reached the maximal effect at 24 h, which was consistent with occludin expression [25]. Therefore, we performed our assessments after cells had been exposed to LPS (10 *μ*g/mL) for 24 h. We next investigated the mechanism by which LPS influenced TJ protein expression.

As an important regulator in signal transduction pathways, MAPK can activate many cell types and stimulate cytokine production during inflammation. Enhanced phosphorylation of p38MAPK and JNK is associated with an LPS-induced decrease of interleukin-10 expression in alveolar macrophages [26]. However, another study reported that LPS injection in alcohol-preferring rats activated p38MAPK but not JNK in rat brain endothelial cells [27], and JNK but not p38MAPK signaling is involved in LPS-mediated ICAM-1 expression in human umbilical vein endothelial cells [28]. In view of these findings, we examined whether p38MAPK and JNK signaling pathways were involved in the LPS-induced decreases of occludin and ZO-1 protein levels in hCMEC/D3 and whether these changes could be reversed by inhibiting p38MAPK and JNK. We found that p38 and JNK phosphorylation were elevated after LPS stimulation, but pretreatment with a p38MAPK or JNK inhibitor before LPS stimulation attenuated phosphorylation of p38 and JNK (Figure 4). These effects were enhanced for occludin but not ZO-1 expression (Figure 3). The novel results of the present study demonstrate that LPS decreased occludin expression in hCMEC/D3 via its effects on the p38MAPK and JNK signaling pathways; however, the precise mechanism by which LPS affects ZO-1 expression remains to be identified. Interestingly, the extent to which occludin mRNA was decreased by LPS treatment was slightly less than that of protein in the current study, indicating that posttranscriptional modifications also played a role.

MMPs, particularly MMP-2, have been shown to mediate BBB disruption and contribute to neuroinflammation [29]. Our results demonstrate that active forms of MMP-2 appear in the supernatant of hCMEC/D3 after LPS stimulation (Figure 5), and inhibitors of both total MMPs and MMP-2 partially attenuated the LPS-induced downregulation of occludin (Figure 6). These results indicate that abnormal increases of MMP-2 in hCMEC/D3 could have influenced the LPS-mediated downregulation of occludin. They also suggest that inhibition of MMP-2 could be an effective strategy to prevent TJ damage due to LPS. MMP-2 belongs to a class of zinc-bound proteases, with functions including induction of inflammation, cleavage of myelin proteins, activation or degradation of disease-modifying cytokines, and direct damage to CNS cells [30]. Abnormal expression and activation of MMPs have been shown to contribute to BBB opening. MMP-2/9 activities lead to decreases in antioxidant and TJ (claudin-5 and occludin) protein levels [31]. We previously demonstrated that exposure of hCMEC/D3 to HIV-infected monocytes resulted in decreased expression of TJ proteins (JAM-A, Occludin, and ZO-1) via modulating MMP-2 and MMP-9 [12]. Therefore, these reports support the hypothesis that MMPs play an important role in regulating TJ protein expression.

MMP-2 is regulated by many kinds of growth and cellular factors through MAPK signaling pathways and is not usually expressed at high levels in normal brain tissue [32, 33]. We previously reported that Ras-ERK and PI3K-Akt are involved in the regulation of HIV-1-induced MMP-9 expression [13], and the decrease in ZO-1 levels mediated by Tat induction was less pronounced in MMP-9-deficient mice compared with wild-type controls [34]. The present study revealed that inhibitors of p38MAPK and JNK markedly abrogated LPS-induced MMP-2 overexpression at both the mRNA and protein levels. LPS might activate MMP-2, which is involved in p38MAPK and JNK signaling, thus causing decreases in TJ protein expression. Here, we present a possible strategy for preventing TJ dysregulation through inhibiting MMP-2 overexpression and abnormal activation of p38MAPK/JNK signaling.

p38MAPK/JNK activation is also associated within LPS regulating c-Fos/c-Jun [35, 36], which then translocates into the nucleus to construct the AP-1 transcription factor [36]. The AP-1 complex is composed of members of three families of DNA-binding proteins: Jun (c-Jun, JunB, v-Jun, and JunD), Fos (Fra-1 and Fra-2, c-Fos, and FosB), and ATF/CREB (ATF1–4, ATF-6, *β*-ATF, and ATFx) [37]. In particular, AP-1 can bind to the enhancer and promoter of MMP-2 in response to a variety of signals MMP-2 expression reportedly requires AP-1; decreased AP-1 binding activity significantly inhibits MMP-2 gelatinolytic activity [38–40]. Taken together, these results strongly suggest that LPS/p38MAPK or JNK/AP-1/MMP-2 signaling may be closely related to metastasis in TJ dysregulation.

## 5. Conclusion

In summary, our findings reveal that LPS-induced dysregulation of TJs in hCMEC/D3, especially occludin, is influenced by upregulated phosphorylation of p38MAPK and JNK, which are also involved in regulating MMP-2 overexpression. Activation of MMP-2 through augmenting p38MAPK/JNK signaling pathways could be one of the mechanisms of LPS-induced dysregulation of TJs in the BBB. All of these events could accelerate the course of secondary brain edema, and further study of MMP-2 and p38MAPK/JNK inhibitors may identify novel therapeutic strategies for patients with CNS infections.
